# UPLC-MS/MS-Based Serum Metabolomics Signature as Biomarkers of Esophagogastric Variceal Bleeding in Patients With Cirrhosis

**DOI:** 10.3389/fcell.2022.839781

**Published:** 2022-03-01

**Authors:** Yingjie Ai, Xiaoquan Huang, Wei Chen, Ling Wu, Siyu Jiang, Ying Chen, Shiyao Chen

**Affiliations:** ^1^ Department of Gastroenterology and Hepatology, Minhang Hospital, Fudan University, Shanghai, China; ^2^ Department of Gastroenterology and Hepatology, Zhongshan Hospital, Fudan University, Shanghai, China

**Keywords:** esophagogastric variceal bleeding, cirrhosis, metabolomics, UPLC-MS/MS, fatty acid, biomarker

## Abstract

**Background**: Esophagogastric variceal bleeding (EVB) is a common and ominous complication of cirrhosis and represents the degree of portal hypertension progression and cirrhosis decompensation, desiderating the investigation into sensitive and specific markers for early detection and prediction. The purpose of this study is to characterize unique metabolites in serum of cirrhotic EVB patients and identify potential noninvasive biomarkers for detecting and assessing risk of variceal bleeding and cirrhosis progression through metabolomics-based approaches and explore possible pathophysiological mechanisms.

**Methods**: We used ultra-performance liquid chromatography coupled to tandem mass spectrometry (UPLC-MS/MS) to profile serum metabolomes. In one discovery cohort (*n* = 26, 13 cases of EVB), univariate and multivariate statistical analyses were performed to demonstrate separation between the two groups and identify differentially expressed metabolites. Potential biomarkers were screened by Boruta and logistic regression analyses, further evaluated by receiver operating characteristic analysis, and tested in two validation cohorts (*n* = 34, 17 cases and *n* = 10, 5 cases).

**Results**: Bioinformatics analyses demonstrated that EVB patients possessed distinct metabolic phenotypes compared with nEVB controls, characterized by seven elevated and six downregulated metabolites, indicating that EVB-related metabolic disturbance might be associated with vitamin metabolism and fatty acid metabolism. Eight potential biomarkers were selected among which citrulline and alpha-aminobutyric acid with moderate AUC values, tested in the validation cohorts, were identified as specific biomarkers of EVB.

**Conclusion**: Our metabolomic study provides an overview of serum metabolic profiles in EVB patients, highlighting the potential utility of UPLC-MS/MS-based serum fingerprint as a feasible avenue for early detection of EVB.

## 1 Introduction

Variceal hemorrhage is one of the most common complications of portal hypertension, representing a leading cause of death in patients with chronic liver disease, especially cirrhosis (Barnett 2018; Kovacs and Jensen 2019; Simonetto et al., 2019). Intrahepatic, splanchnic, and systemic hemodynamic alterations, resulting from both enhanced resistance to portal flow and increased portal venous blood inflow, lead to portal hypertension (PHT) (Iwakiri 2014). Gastroesophageal varices are the most important portosystemic collaterals due to their propensity to rupture, and acute bleeding is life-threatening, requiring inpatient care (Garcia-Tsao and Bosch 2010). Esophageal and gastric variceal bleeding is associated with a high morbidity and mortality rate despite accumulating diagnostic and therapeutic developments in the management of acute episodes recently (Sharara and Rockey 2001; Garcia-Tsao and Bosch 2010; Ibrahim et al., 2018). Gastroesophageal varices are prevalent and present in about 67% of cirrhotic patients in a previous study (D'Amico et al., 2014), and their incidences gradually increase from 5% at 1 year to 28% at 3 years (Merli et al., 2003). The 1-year rate of a first variceal hemorrhage is approximately 12% (North Italian Endoscopic Club for the Study and Treatment of Esophageal Varices 1988), and patients suffering rebleeding within 1 year account for over 60% without optimal intervention (Bosch and García-Pagán 2003). Hence, there is an urgent need for better noninvasive clinical biomarkers to predict bleeding episodes in the early stage.

Recently, detection and diagnosis for EVB (esophagogastric variceal bleeding) mostly depend on clinical, endoscopic, and other techniques with several shortcomings (Bosch and García-Pagán 2003; Groszmann et al., 2005; Garcia-Tsao et al., 2007; Seo 2018). Measurement of the hepatic venous pressure gradient (HVPG) which helps estimate the risk of bleeding remains the gold standard for diagnosing PHT, although it is invasive and demanding. Endoscopy can be applied for demonstrating the presence of varices and bleeding, which is, however, also invasive, expensive, and uncomfortable (Garcia-Tsao and Bosch 2010; Seo 2018). Considering that most of these existing measures require surgical operation, it is essential to explore noninvasive, cheap, and convenient preventive screening and detection methods to assess the severity of PHT and risk of variceal bleeding.

Metabolomics, focusing on investigation of biochemical processes associated with metabolites, is a prospective research field downstream of transcriptomics, epigenomics, genomics, and proteomics. Notably, as metabolites are present in available biofluids and easy to acquire, metabolite-based strategies have been increasingly applied, including the use of markers for clinical diagnosis especially in tumors such as colorectal cancer (Yachida et al., 2019; Shen et al., 2020), gastric cancer (Erawijantari et al., 2020), and esophageal cancer (Sun et al., 2019). Thus, it is highly attractive to identify EVB-related metabolites discriminatory for biological perturbations and further probe predictive biomarkers, but surprisingly, very few metabolomic studies have been performed in EVB so far. Liquid chromatography–tandem mass spectrometry (UPLC-MS/MS) is a powerful approach for detecting metabolomics differences derived from disease-associated alterations in biological fluids (Huang et al., 2018; Ly et al., 2020; Xie et al., 2021). Enhanced separation efficiency and sufficient resolution enables UPLC-MS/MS to be an appropriate method for creation of diagnostic metabolic profiles and identification of candidate biomarkers.

Here, we applied an integrated approach combining liquid chromatography–mass spectrometry (UPLC-MS) and mass spectrometry (MS) to analyze the serum metabolome obtained from EVB patients and nEVB patients and document EVB-specific metabolic profiles. Our study discovered a significant difference between the serum metabolome of two groups and underlined the potential role of an omics approach in understanding metabolic pathways involved in EVB, proposing that the UPLC-MS/MS-based serum metabolomics fingerprints could be utilized as predictors of EVB.

## 2 Methods

### 2.1 Ethical Consideration

The study protocol was approved by the Ethics Committee of Zhongshan Hospital of Fudan University (B2015-133R) and Minhang Hospital of Fudan University (2018-009-01X), and all the procedures were conducted in accordance with the Declaration of Helsinki. Written informed consent was obtained from each participant prior to taking part in the study.

### 2.2 Patients

**FIGURE 1:**
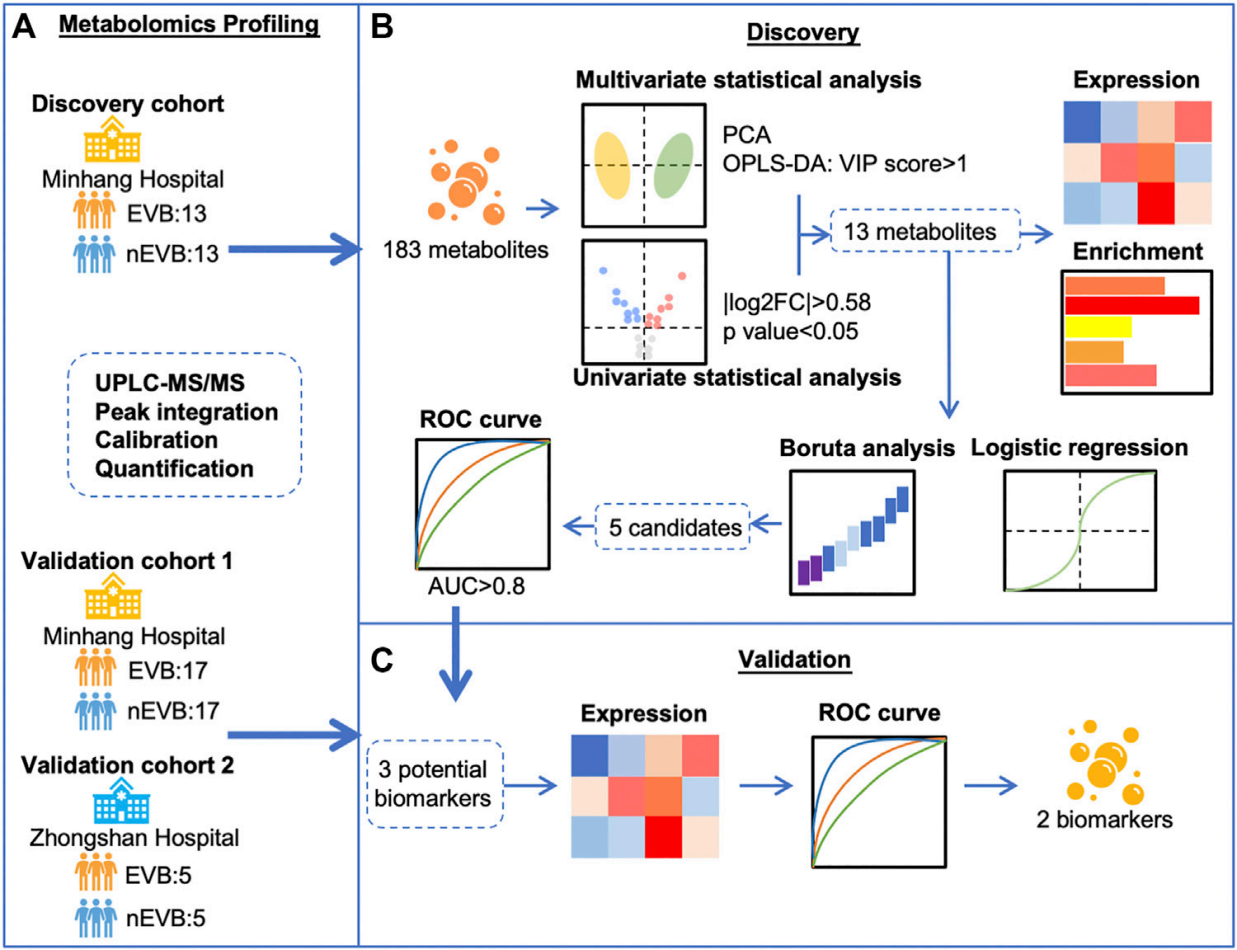
Schematic flow chart for the identification of EVB biomarkers in patients with cirrhosis. **(A)** Metabolomics profiling of serum samples from three cohorts including 35 EVB and 35 nEVB cirrhotic patients was obtained through UPLC-MS/MS. **(B)** Thirteen metabolites were filtered by different statistical analyses in the discovery cohort and analyzed by expression level, functional enrichment, logistic regression, and Boruta analysis. **(C)** Validation cohorts were used to verify the change of metabolite expression derived from EVB and test their predictive accuracy to finally identify two biomarkers.

We recruited three cohorts to investigate specific metabolic alteration associated with EVB and evaluate possible biomarkers (Figure 1). Twenty-six cirrhotic patients (13 EVB and 13 nEVB) were enrolled in the discovery cohort from March 2018 to January 2019 at Minhang Hospital, Fudan University, while 34 cirrhotic patients (17 EVB and 17 nEVB) were enrolled in the validation cohort 1 from May 2018 to December 2021 at the same hospital. In addition, 10 cirrhotic patients (five EVB and five nEVB) were enrolled in the validation cohort 2 from June 2017 to September 2018 at Zhongshan Hospital of Fudan University.

Patients with liver cirrhosis complicated with esophagogastric varices were included, while patients who experienced bleeding, fasting, infection, and antibiotic use within 2 weeks and patients admitted from the emergency department were excluded. The patients were divided into nEVB and EVB groups according to whether they had EVB episodes in the past.

Bleeding episodes were defined as the presence of hematemesis and/or melena. For patients with hematemesis and/or melena, upper endoscopy was performed, and subsequent endoscopic treatment was performed when necessary.

All blood samples were extracted on the second day of admission and in the morning after a 10-h fasting period but before endoscopic treatment. All patients were fed routinely without special nutrition or feeding tubes.

### 2.3 Blood sample collection and UPLC-MS/MS

We extracted 3–4 ml blood from participants, injected it into special separation tubes for serum, and mixed it immediately. The tubes were placed at room temperature for about 30 min until the blood completely coagulated and then centrifuged for 10 min at 1,000 g. The upper transparent liquid was separated for testing. An ultraperformance liquid chromatography coupled to tandem mass spectrometry (UPLC-MS/MS) system (ACQUITY UPLC-Xevo TQ-S, Waters Corp., Milford, MA, United States) was used to quantitate all targeted metabolites and performed by Metabo-Profile (Shanghai, China). The derivatized samples and serial dilutions of derivatized stock standards were randomly analyzed and quantitated by UPLC-MS/MS. 310 of standard substances, including 12 subclasses, were obtained from Sigma-Aldrich (St. Louis, MO, United States), Steraloids Inc. (Newport, RI, United States), and TRC Chemicals (Toronto, ON, Canada). Three types of quality control samples, that is, test mixtures, internal standards, and pooled biological samples are routinely used in the metabolomics platform. The derivatized pooled quality control samples were injected in every 14 test samples. Raw data generated by UPLC-MS/MS were processed using QuanMET (v2.0, Metabo-Profile, Shanghai, China) to perform peak integration, calibration, and quantification for each metabolite.

### 2.4 Metabolomics Data Analysis

To establish a global overview of the differential characteristics of the EVB patients relative to nEVB controls, multivariate data analysis was applied to the UPLC-MS/MS data. Principal component analysis (PCA) and orthogonal partial least squares–discriminant analysis (OPLS-DA) was utilized on UPLC-MS/MS processed data to reduce dimension and maximize class differences by the ropls R package. First, a PCA model was used to examine an overview trend and outliers in the metabolic pattern. Data were visualized by means of PC score plots, where each point represents an individual sample. OPLS-DA was applied to unit variance and distinguished the differences of serum profiles between two groups, and VIP (variable importance for the projection) values are calculated to summarize the importance of each variable for the OPLS-DA model.

Univariate data analysis was performed to select differentially expressed metabolites and presented by the ggplot2 R package. When presented *via* a boxplot, the expression/concentration (EXP) is processed as y = log2 (EXP+1). The Boruta algorithm and logistic regression model were implemented for biomarker identification by Boruta and ResourceSelection R package, respectively. Receiver operating characteristic analysis (ROC) performed by the pROC R package evaluated specificity and sensitivity of candidate biomarkers. Functional enrichment was built using MetaboAnalyst 5.0. The compounds are enriched according to the Human Metabolome Database (HMDB).

### 2.5 Clinical Statistical Analysis

Continuous variables with normal distribution were presented as mean ± standard deviation (SD) or median with interquartile range (IQR) when the distribution of the values significantly deviated from the normal distribution. The categorical variables were expressed as number and proportion. All statistical analyses were performed by SPSS 26.0 (IBM, Armonk, NY, United States) and R 4.0.3.

## 3 Results

### 3.1 Demographic Data Analysis and Serum Metabolic Profiles

Three cohorts consisting of total 70 participants with cirrhosis were enrolled in this study as the discovery cohort includes 13 EVB patients and 13 nEVB controls, while the validation cohort 1 from the same center as the discovery cohort includes 17 EVB patients and 17 nEVB controls, and the validation cohort 2 from a different center includes five EVB patients and five nEVB controls. Their main characteristics are shown in Table 1, Supplementary Tables S1, S2 respectively. There was no significant difference in the prevalence of general factors such as age, gender, height, weight, and BMI; basic diseases including hypertension, diabetes, and coronary heart diseases; cirrhosis-related factors including cirrhosis etiology and Child–Pugh score; and also biochemical indicators reflecting metabolism such as TC, TG, fasting plasma glucose, LDL, and HDL-c.

**TABLE 1:** Demographics and clinical characteristics of EVB patients and nEVB controls in the discovery cohort.

Characteristics	nEVB (n = 13)	EVB (n = 13)	*p* Value
Age, mean (SD), years	66.08 (7.85)	64.69 (13.86)	0.757
Gender, n (%)			1.000
Male	7 (53.80)	7 (53.80)	
Female	6 (46.20)	6 (46.20)	
Weight, mean (SD), kg	63.19 (9.74)	59.89 (7.91)	0.351
Height, mean (SD), cm	160.31 (5.84)	163.69 (6.25)	0.166
BMI, mean (SD), kg/m^2^	24.60 (3.58)	22.28 (2.18)	0.058
Cirrhosis etiology, n (%)			0.731
Alcohol	2 (15.40)	3 (23.10)	
Hepatitis B	5 (38.50)	6 (46.2)	
Schistosomiasis	3 (23.10)	3 (23.10)	
Other	3 (23.10)	1 (7.70)	
CHILD score, mean (SD)	7.46 (1.85)	6.62 (1.19)	0.182
Albumin, mean (SD), g/L	33.39 (4.96)	33.69 (3.97)	0.863
Total bilirubin, median (range), mmol/L	20.20 [10.65–44.35]	21.10 [14.75–26.90]	0.724
PT, median (range), s	13.70 [12.50–14.35]	14.40 [13.30–15.80]	0.169
HDL-c, mean (SD), mmol/L	0.99 (0.41)	0.93 (0.45)	0.754
LDL, mean (SD), mmol/L	1.81 (0.63)	1.98 (0.97)	0.610
Fasting plasma glucose, mean (SD), mmol/L	5.95 (1.77)	5.57 (1.16)	0.518
TC, mean (SD), mmol/L	3.56 (2.02)	3.11 (1.11)	0.490
TG, mean (SD), mmol/L	0.98 (0.37)	0.99 (0.36)	0.937
Hepatic encephalopathy, n (%)			0.480
Yes	2 (15.40)	0 (0.00)	
No	11 (84.60)	13 (100.00)	
PVT, n (%)			1.000
Yes	2 (15.40)	3 (23.10)	
No	11 (84.60)	10 (76.90)	
Splenectomy, n (%)			1.000
Yes	2 (15.40)	3 (23.10)	
No	11 (84.60)	10 (76.90)	
Ascites, n (%)			0.691
Yes	7 (53.80)	8 (61.50)	
Hypertension, n (%)	6 (46.20)	5 (38.50)	
Yes	4 (30.80)	6 (46.20)	
No	9 (69.20)	7 (53.80)	
Diabetes, n (%)			1.000
Yes	7 (53.80)	6 (46.20)	
No	6 (46.20)	7 (53.80)	
CAD, n (%)			1.000
Yes	1 (7.70)	0 (0.00)	
No	12 (92.30)	13 (100.00)	

Metabolomic profiles of serum samples were acquired through UPLC-MS/MS to get a global overview of metabolic alteration derived from EVB, and 183 metabolites were successfully identified. All detected metabolites could be classified into 17 categories, among which amino acids, carbohydrates, fatty acids, and organic acids accounted for the majority (Supplementary Figure S1).

### 3.2 Candidate Metabolite Selection

**FIGURE 2:**
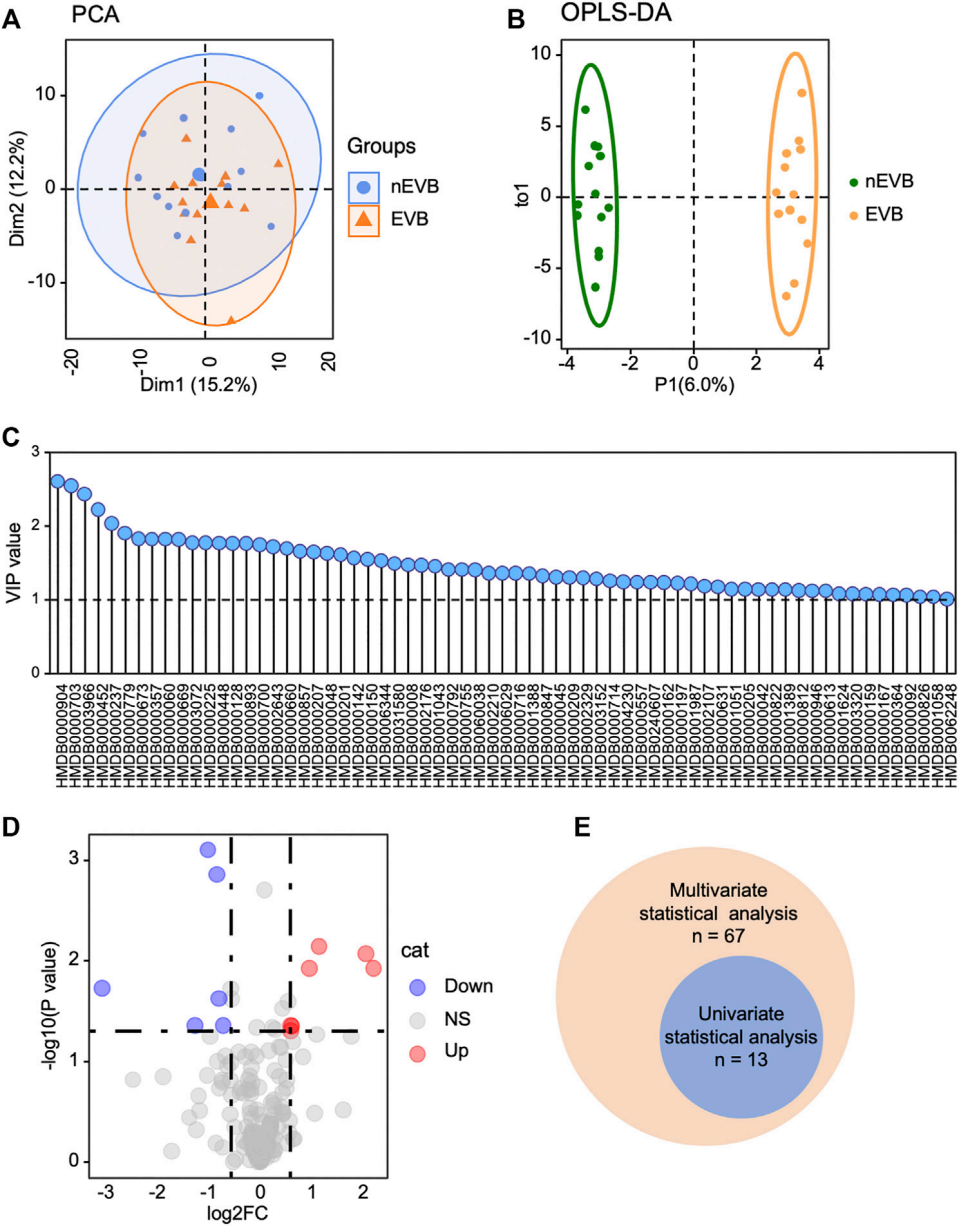
Metabolic profile of EVB patients is distinguished from that of nEVB controls. **(A–B)** PCA and OPLS-DA plot demonstrated metabolic difference between two groups. **(C)** Multivariate statistical analysis screened 67 metabolites with a VIP score of 1.0. **(D)** EVB group is characterized by seven increased and six decreased metabolites with a threshold of |log2FC|> 0.58 and *p* < 0.05. **(E)** Venn diagram exhibited the intersection of selected metabolites generated from multivariate and univariate statistical analyses.

To reveal metabolic changes in EVB patients, multivariate statistical analysis was applied. Initially, unsupervised principal component analysis (PCA) demonstrated that EVB patients displayed a similar but also slightly different metabolic phenotype compared with nEVB controls, and the two groups are mostly overlapping and partially separated, with 15 and 12% variation explained by principal component PC1 and PC2, respectively (Figure 2A). However, considering the complexity of metabolomics, PCA analysis may not be able to distinguish samples of different groups well. Therefore, to further optimize the difference, orthogonal partial least squares–discriminant analysis (OPLS-DA), a supervised modeling method, was then utilized to visualize the alteration. The samples from two groups were separable according to the score scatter plot with one predictive component and one orthogonal component (R2Xcum = 0.423, R2Ycum = 0.992, Q2Ycum = 0.348, Figure 2B). These results suggested that serum metabolome alteration can be used to discriminate EVB patients from nEVB controls. The fluctuating metabolites were screened according to the significance of their contribution to the model which was quantified by the variable importance on projection (VIP). 36.6% (*n* = 67) of measured analytes were picked out based on a threshold of VIP >1 (Figure 2C).

The detected metabolites were also filtered by the following standards, |log2FC|> 0.58 and *p* < 0.05, and 13 of them were differentially expressed (Figure 2D). The intersection of selected metabolites generated from multivariate and univariate statistical analyses was considered to be candidate biomarkers (Figure 2E), listed in Table 2.

**TABLE 2:** Selected candidate biomarkers.

HMDB	Metabolite	Class	VIP	log2FC	*p value*	Change
HMDB0000893	Suberic acid	Fatty acids	1.759	0.947	0.012	Up
HMDB0003072	Quinic acid	Organic acids	1.770	-3.074	0.019	Down
HMDB0000237	Propionic acid	SCFAs	2.031	-0.724	0.044	Down
HMDB0000857	Pimelic acid	Fatty acids	1.652	0.607	0.045	Up
HMDB0000048	Melibiose	Carbohydrates	1.627	0.586	0.044	Up
HMDB0000703	Mandelic acid	Benzenoids	2.543	-1.018	0.001	Down
HMDB0000197	Indoleacetic acid	Indoles	1.220	-1.267	0.044	Down
HMDB0000904	Citrulline	Amino acids	2.602	-0.842	0.001	Down
HMDB0000452	alpha-aminobutyric acid	Amino acids	2.220	1.136	0.007	Up
HMDB0000448	Adipic acid	Fatty acids	1.765	2.197	0.012	Up
HMDB0000201	Acetylcarnitine	Carnitines	1.608	0.589	0.050	Up
HMDB0000357	3-hydroxybutyric acid	Organic acids	1.821	2.044	0.009	Up
HMDB0001987	2-hydroxy-2-methylbutyric acid	Organic acids	1.212	-0.809	0.024	Down

### 3.3 Metabolite Expression and Function Enrichment

**FIGURE 3:**
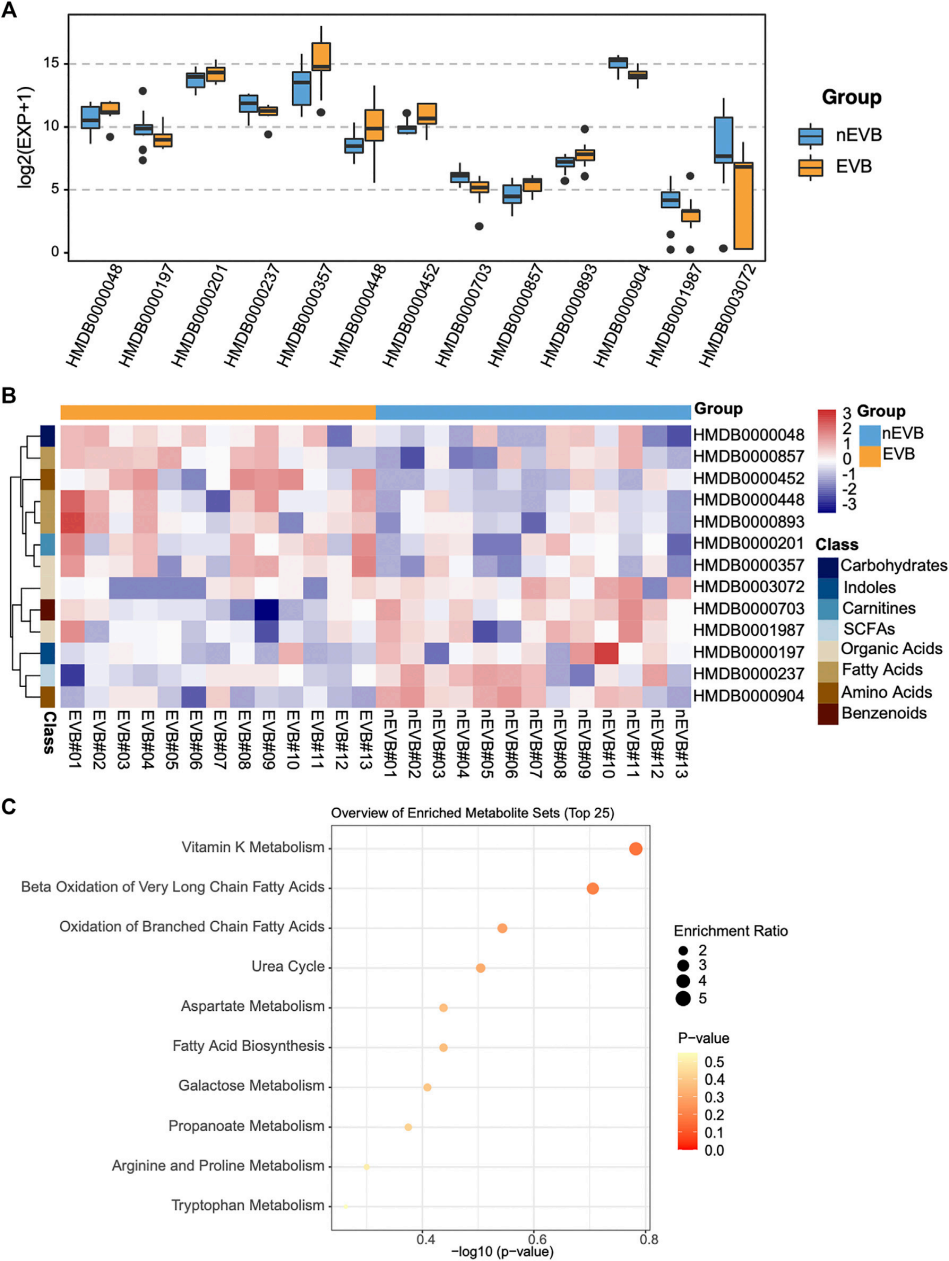
Thirteen candidate metabolites were aberrantly expressed in EVB samples compared with nEVB controls. **(A)** Expression of selected metabolites between EVB and nEVB groups. **(B)** Heatmap of differentially expressed metabolites. **(C)** Bubble pattern of top 25 metabolite sets with enrichment ratio and *p* value.

Compared with nEVB controls, seven metabolites were upregulated in EVB patients, namely, acetylcarnitine, 3-hydroxybutyric acid, adipic acid, alpha-aminobutyric acid, pimelic acid, suberic acid, and melibiose, while the remaining six analytes were downregulated, including indoleacetic acid, propionic acid, mandelic acid, citrulline, a-hydroxy-2-methylbutyric acid, and quinic acid (Figures 3A,B). A large part of these aberrantly expressed metabolites were organic acids and fatty acids (Figure 3B).

To obtain an overview of variations in metabolic pathways of EVB patients, which might contribute to explain the mechanism of disease, metabolite set enrichment analysis was performed. Differentially expressed metabolites in EVB patients were mainly related to vitamin K metabolism, and more importantly, fatty acid metabolism as Beta Oxidation of Very Long Chain Fatty Acids, Oxidation of Branched Chain Fatty Acids, and Fatty Acid Biosynthesis were all enriched (Figure 3C).

### 3.4 Biomarker Identification and Diagnosis Evaluation

**FIGURE 4:**
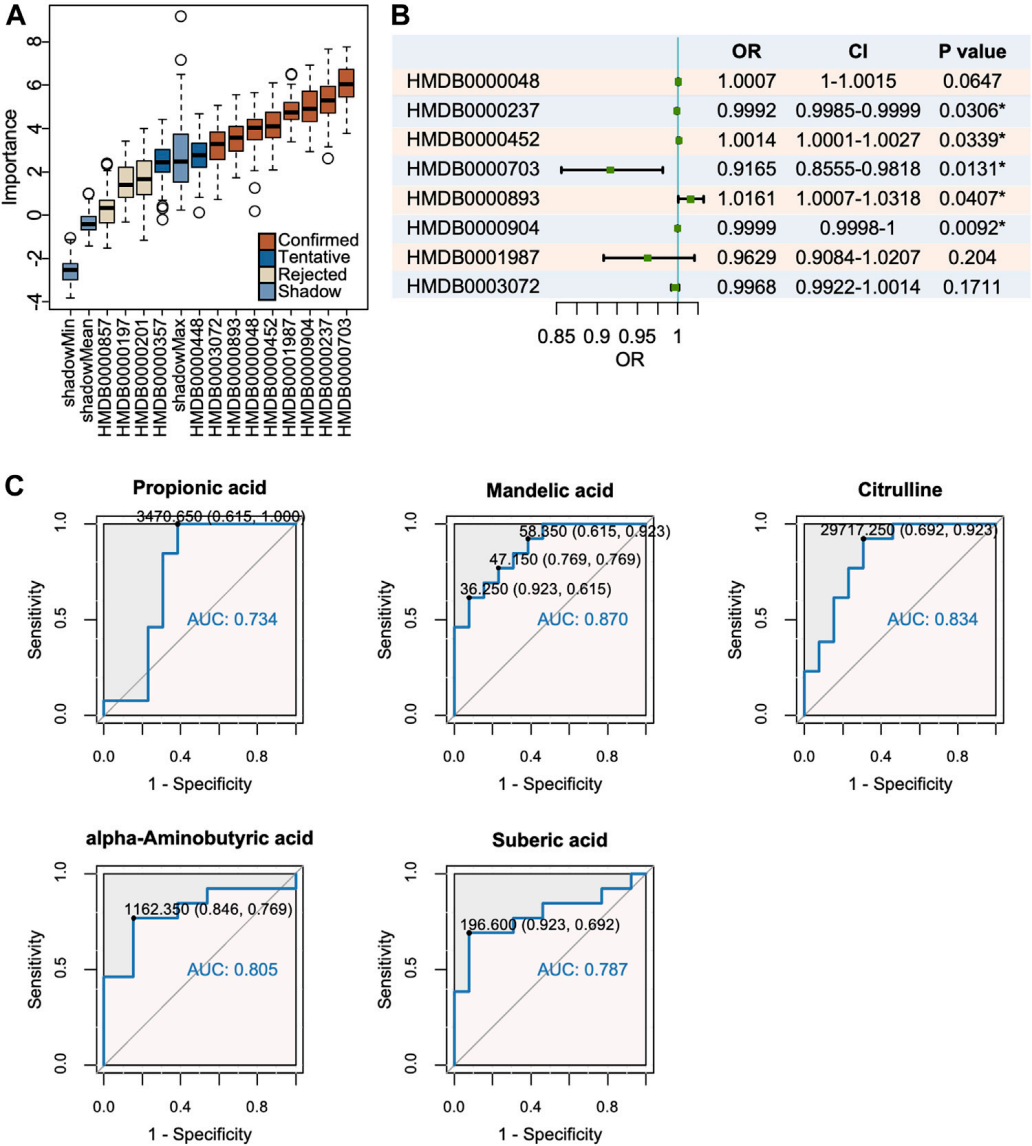
Mandelic acid, citrulline and alpha-aminobutyric acid were identified as potential biomarkers. **(A)** The Boruta analysis certified 8 metabolites as confirmed candidates. **(B)** The logistic regression model of candidate metabolites. **(C)** Mandelic acid, citrulline and alpha-aminobutyric acid were selected with a threshold of AUC >0.80.

Machine learning methods are often applied for classification problem, among which the Boruta algorithm, a system for feature selection, is outstanding in identification of truly important variables in the full set. Boruta analysis determined eight metabolites as “confirmed” and the remaining as “rejected” or “tentative” (Figure 4A). The logistic regression model further excluded several candidates with a threshold of *p* < 0.05 and identified the remaining five as possible biomarkers (Figure 4B). To evaluate their diagnostic accuracy, selected metabolites were then assessed by the ROC, among which those with AUC>0.8 were as follows: mandelic acid, citrulline, and alpha-aminobutyric acid (Figure 4C).

### 3.5 External Validation

**FIGURE 5:**
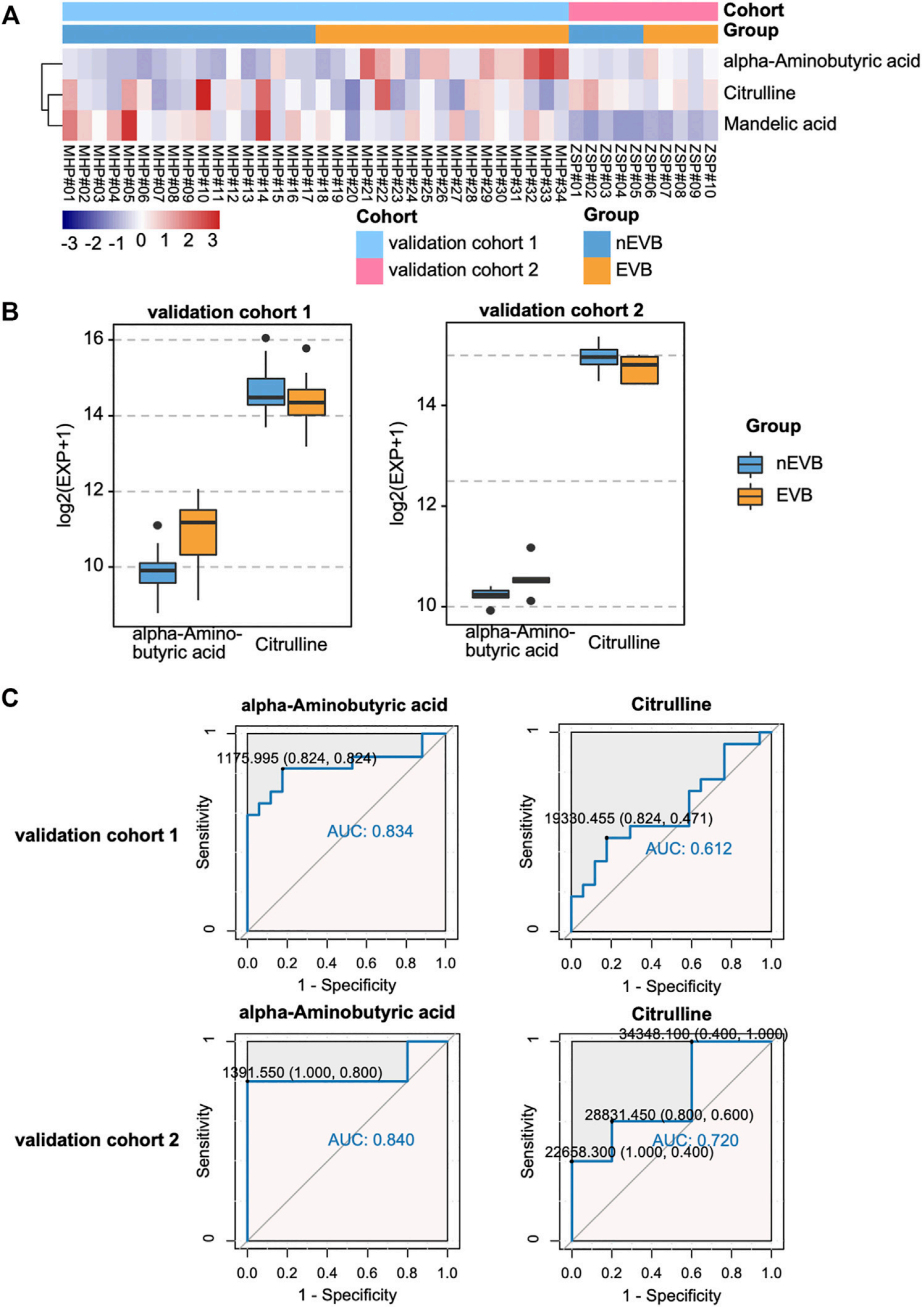
Diagnostic value of potential biomarkers in both validation cohorts. **(A–B)** Expression of mandelic acid, citrulline, and alpha-aminobutyric acid between the two groups. **(C)** ROC plot for EVB occurrence.

To assess the prognostic role of this metabolic signature, we applied three potential biomarkers to 34 patients in the validation cohort 1 and 10 patients in the validation cohort 2. Among them, only citrulline and alpha-aminobutyric acid exhibited identical significant expression difference between two groups in the discovery and both validation cohorts (Figures 5A,B). The ROC curve demonstrated that alpha-aminobutyric acid and citrulline showed moderate reliability in both validation cohort 1 (AUC = 0.834 and 0.612) and validation cohort 2 (AUC = 0.840 and 0.720), indicating a fair diagnostic value of both metabolites for EVB in patients with cirrhosis (Figure 5C).

## 4 Discussion

EVB in patients with cirrhosis indicates significant portal hypertension and predicts decompensation (D'Amico et al., 2014). Nonselective beta-blockers are effective in preventing bleeding episodes and development of decompensating events, but once varices rupture, acute bleeding can be life-threatening and need multiple management strategies, including vasoactive drugs and endoscopic and radiological intervention (Garcia-Tsao and Bosch 2010). Furthermore, although continuously optimized treatment improves patient survival, the mortality rate of 6 weeks is still up to 20% (Ibrahim et al., 2018). The methods to predict or detect hemorrhage at the early stage may help alleviate terrible prognosis and need to be discovered urgently. Studies have shown that changes of molecular and biochemical metabolism are prior to the morphologic and functional alterations and can be possible precursors for disease (Wilson et al., 2005; Martins et al., 2006; Johnson et al., 2016). Hence, metabolomics identifying the characteristic metabolic phenotype enables early recognition of pathological changes and provides possibility of timely diagnosis and then intervention. Metabolomics has been widely applied in a great disease spectrum including various liver diseases such as decompensated cirrhosis (McPhail et al., 2016), hepatocellular carcinoma (Ladep et al., 2014), and inflammatory bowel disease (Williams et al., 2009) and also complications of cirrhosis such as minimal hepatic encephalopathy (Jiménez et al., 2010). However, still no studies involve EVB and shape a global overview of its metabolic signature through combined technologies.

In this study, by analyzing the serum metabolome of EVB and nEVB cirrhotic patients through UPLC-MS/MS, we acquired both accurate prediction and mechanistic insights into metabolic perturbations derived from EVB, characterized by vitamin and fatty acid metabolism. For the first time, we identified citrulline and alpha-aminobutyric acid as possible prognostic markers but still need further validation in more cohorts.

UPLC-MS/MS-based metabolomics provides chances to get a special insight into the pathogenesis of the disease and opportunities to search for potential biomarkers based on disease-associated metabolic alterations. Metabolomics permits an interesting perspective into tracing biochemical alteration/evolution which develops with transition from a healthy liver to a cirrhotic organ and is further decompensated (Johnson et al., 2016; McPhail et al., 2016). Cirrhosis, mostly generated from long-term alcohol intake, viral infection, and increasing obesity and diabetes, is mainly characterized by disorder of lipid, amino acid, and carbohydrate homeostasis (Dietrich et al., 2016). EVB patients in our study demonstrated dysregulated metabolites as follows: increased quantities of acetylcarnitine, 3-hydroxybutyric acid, adipic acid, alpha-aminobutyric acid, pimelic acid, suberic acid, and melibiose and decreased levels of indoleacetic acid, propionic acid, mandelic acid, citrulline, a-hydroxy-2-methylbutyric acid, and quinic acid. Acetylcarnitine, involved in oxidation of branched chain fatty acids, was also reported to be upregulated in serum metabolomes of cirrhotic patients (Beyoğlu and Idle 2013), while downregulated citrulline was, likewise, observed in hepatocellular carcinoma cases (Chen et al., 2011). Besides, phenyllactic acid was recognized as a conserved biomarker for alcohol-induced liver disease through UPLC-MS-based urine metabolomics (Manna et al., 2011). Several previous studies showed that aggravated aerobic glycolysis indicated involvement of disturbed carbohydrate metabolism in hepatic fibrosis (Beyoğlu and Idle 2013; Du et al., 2018) and loss of vitamin A–containing lipid droplets and serum lipids suggested that cirrhosis was associated with vitamin and lipid metabolic alterations (Geubel et al., 1991; Simbrunner et al., 2020), consistent with our findings of EVB-related metabolism profiles. The highlighted metabolic pathways might depict the pathophysiological changes and metabolic disturbances in the disease progression and allow potential strategies to target themselves as accesses to treat cirrhosis (Johnson et al., 2016). However, detailed intervention and the underlying mechanism require further exploration.

Alpha-aminobutyric acid and citrulline were identified as a prospective biomarker for EVB after multiple filtrations. Citrulline, reported to be significantly decreased in the serum of HCC patients, is engaged in urea cycle and takes charge of disposal of excess nitrogen in hepatocytes, coinciding with a proteomic study of downregulated urea cycle enzymes and activities in HCC cell lines (Chen et al., 2011). Meanwhile, several research studies discussed about inhibiting fat accumulation of citrulline by improving lipid metabolism in nonalcoholic fatty liver diseases (Kudo et al., 2021). Therefore, we hypothesized that changes of proteins or genes regulating urea cycle or lipid metabolism might also occur during the initiation and progression of EVB, which could be checked by proteomics or transcriptomics in later research studies. Oral citrulline supplementation has been widely applied in research studies about NAFLD (Sellmann et al., 2017), so it is feasible to investigate whether citrulline contributes to alleviation of cirrhosis and decrease of complications such as EVB. As to alpha-aminobutyric acid, increased concentration has been detected in patients with liver disease and alcoholism (Beyoğlu and Idle 2013), but it is somehow not prevalent and convenient for measurement. A recent report suggested that alpha-aminobutyric acid could predict the clinical outcome of pediatric acute liver failure and help make decisions for liver transplantation (Rudnick et al., 2009). As a few studies showed that enhanced ABA reflected liver injury in patients with sepsis and MODS (Effros 2011), ABA potentially participates in liver diseases and is worth more investigation.

The major limitation of this study is the relatively small sample size, which leads to an ignorable impact of individual differences on the metabolic profiles. Therefore, more patients should be included in future research. Besides, serum metabolomes could be affected by numerous factors, such as food intake and disease status, and it is impractical to standardize all related factors of every individual. Since we are pursuing common metabolic differences caused by EVB, methods to minimize the bias of diets and metabolism-related diseases should be found and applied. In addition, larger datasets could be established to examine the predictive effect of selected biomarkers, and follow-up could be extended to test the value of metabolites on variceal bleeding–associated mortality and rebleeding morbidity.

## 5 Conclusion

In summary, UPLC-MS/MS coupled with data analysis initially demonstrated that the characteristic metabolic profiles in EVB cirrhotic patients were partially distinct from those in nEVB controls without a history of variceal bleeding and screened out citrulline and alpha-aminobutyric acid as biomarkers. The altered serum metabolites potentially revealed disruption of lipid metabolism, which could be possibly related with initiation and progression of portal hypertension and may deepen our understanding of molecular mechanisms underlying pathogenesis in cirrhosis.

## Data Availability

The data presented in the study are deposited in the MetaboLights repository, accession number MTBLS4252 (www.ebi.ac.uk/metabolights/MTBLS4252).
