# Supplementary figures and images for: UPLC-MS/MS-Based Serum Metabolomics Signature as Biomarkers of Esophagogastric Variceal Bleeding in Patients With Cirrhosis
Source: Front Cell Dev Biol. 2022 Mar 1;10:839781. doi: 10.3389/fcell.2022.839781 (PMC8922031; doi:10.3389/fcell.2022.839781)

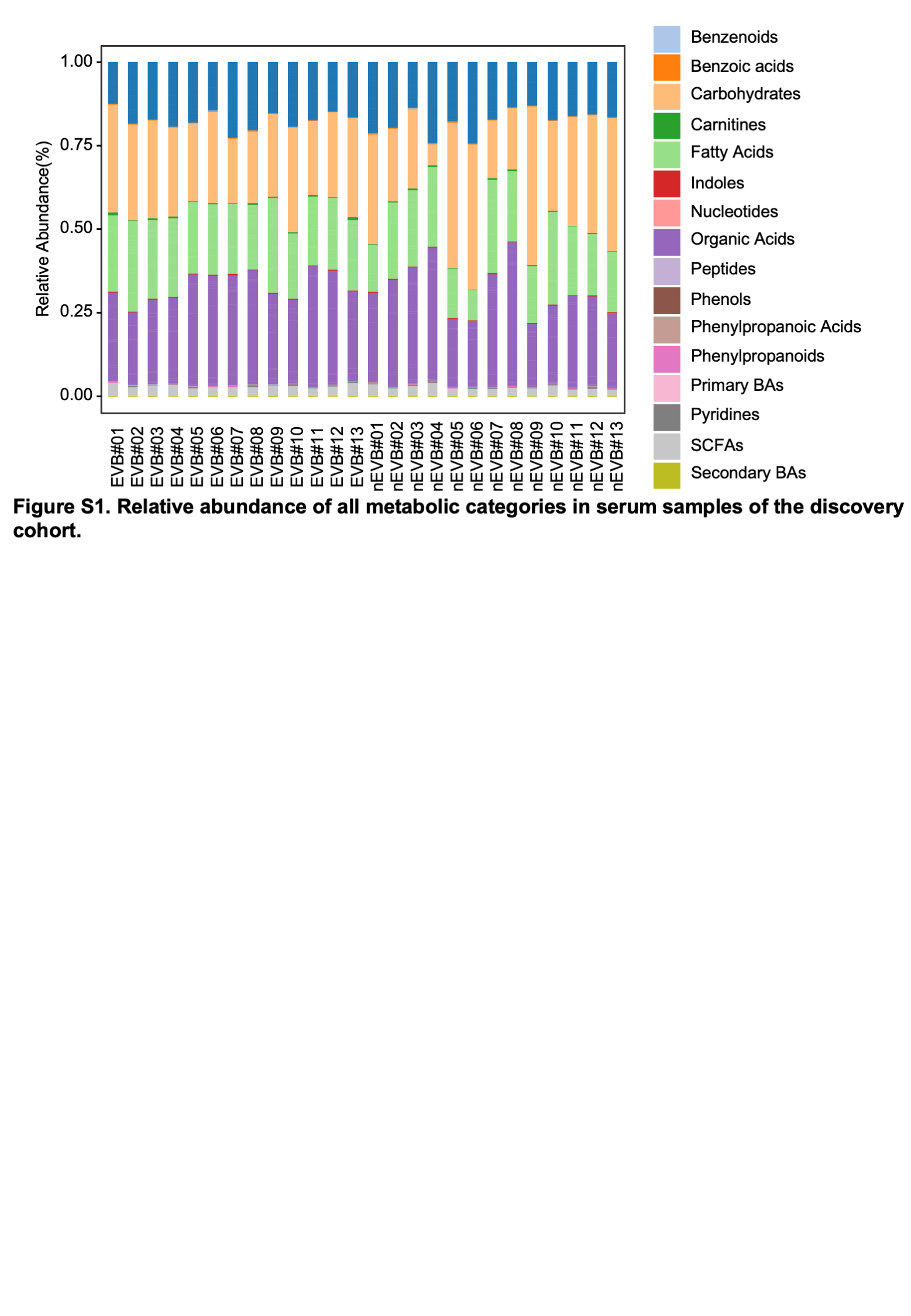

Supplement: Supplementary file 1 [file Image1.tiff]
