# Supplementary material for: UPLC-MS/MS-Based Serum Metabolomics Signature as Biomarkers of Esophagogastric Variceal Bleeding in Patients With Cirrhosis
Source: Front Cell Dev Biol. 2022 Mar 1;10:839781. doi: 10.3389/fcell.2022.839781 (PMC8922031; doi:10.3389/fcell.2022.839781)
Supplement: Supplementary file 2 [file Table1.docx]

**Table S1** Demographics and clinical characteristics of EVB patients and nEVB controls in the validation cohort 1

| Characteristic | nEVB (n=17) | EVB (n=17) | P value |
| --- | --- | --- | --- |
| Age, mean (SD), years | 55.53(15.62) | 53.71(11.30) | 0.699 |
| Gender, n (%) |  |  | 0.072 |
| Male | 12(70.6) | 16(94.1) |  |
| Female | 5(29.4) | 1(5.9) |  |
| Weight, mean (SD), kg | 64.41(10.74) | 70.94(9.59) | 0.043 |
| Height, mean (SD), cm | 167.12(9.38) | 169.18(5.20) | 0.436 |
| BMI, mean (SD), kg/m^2^ | 23.02(3.04) | 24.88(2.63) | 0.055 |
| Cirrhosis etiology, n (%) |  |  | 0.579 |
| Alcohol | 1(5.9) | 4(23.5) |  |
| Hepatitis B | 10(59.8) | 10(59.8) |  |
| Alcohol + Hepatitis B | 1(5.9) | 1(5.9) |  |
| Schistosomiasis | 3(17.6) | 1(5.9) |  |
| Schistosomiasis + Hepatitis B | 1(5.9) | 0(0.0) |  |
| Other | 1(23.10) | 1(7.70) |  |
| CHILD score, mean (SD) | 6.76(1.86) | 6.71(2.02) | 0.930 |
| Albumin, mean (SD), g/L | 35.29(7.34) | 37.12(6.56) | 0.450 |
| Total bilirubin, median (range), mmol/L | 20.40[13.60-34.40] | 24.40[16.55-34.45] | 0.730 |
| PT, mean (SD), s | 15.08(2.24) | 14.83(2.52) | 0.759 |
| Splenectomy, n (%) |  |  | 0.671 |
| Yes | 3(17.6) | 4(23.5) |  |
| No | 14(82.40) | 13(76.5) |  |
| Ascites, n (%) |  |  | 0.732 |
| Yes | 9(52.9) | 8(47.1) |  |
| No | 8(47.1) | 9(52.9) |  |
| Hepatic encephalopathy, n (%) |  |  | 0.545 |
| Yes | 2(11.8) | 1(5.9) |  |
| No | 15(88.2) | 16(94.1) |  |
| PVT, n (%) |  |  | 0.714 |
| Yes | 5(29.4) | 6(35.3) |  |
| No | 12(70.6) | 11(64.7) |  |
| Hypertension, n (%) |  |  | 0.714 |
| Yes | 6(35.3) | 5(29.4) |  |
| No | 11(64.7) | 12(70.6) |  |
| Diabetes, n (%) |  |  | 0.106 |
| Yes | 6(11.8) | 12(35.3) |  |
| No | 15(88.2) | 11(64.7) |  |
| CAD, n (%) |  |  |  |
| Yes | 17(100.0) | 0(0.0) |  |
| No | 17(100.0) | 0(0.0) |  |
