# Supplementary material for: UPLC-MS/MS-Based Serum Metabolomics Signature as Biomarkers of Esophagogastric Variceal Bleeding in Patients With Cirrhosis
Source: Front Cell Dev Biol. 2022 Mar 1;10:839781. doi: 10.3389/fcell.2022.839781 (PMC8922031; doi:10.3389/fcell.2022.839781)
Supplement: Supplementary file 3 [file Table2.docx]

**Table S2** Demographics and clinical characteristics of EVB patients and nEVB controls in the validation cohort 2

| Characteristic | nEVB (n=5) | EVB (n=5) | P value |
| --- | --- | --- | --- |
| Age, mean (SD), years | 54.13 (4.82) | 46.92 (6.40) | 0.079 |
| Gender, n (%) |  |  | 0.093 |
| Male | 2(40.00) | 4(80.00) |  |
| Female | 3(60.00) | 1(20.00) |  |
| Weight, mean (SD), kg | 59.00(4.18) | 61.40(13.75) | 0.720 |
| Height, mean (SD), cm | 159.60(5.90) | 162.40(8.30) | 0.556 |
| BMI, mean (SD), kg/m^2^ | 23.19(1.70) | 23.11(4.22) | 0.969 |
| Cirrhosis etiology, n (%) |  |  | 1.000 |
| Hepatitis B | 4(80.00) | 4(80.00) |  |
| Hepatitis C | 1(20.00) | 1(20.00) |  |
| CHILD score, mean (SD) | 6.80(1.79) | 6.00(0.00) | 0.347 |
| Splenectomy, n (%) |  |  | 1.000 |
| Yes | 2(40.00) | 2(40.00) |  |
| No | 3(60.00) | 3(60.00) |  |
| Ascites, n (%) |  |  | 1.000 |
| Yes | 1(20.00) | 0(0.00) |  |
| No | 4(80.00) | 5(100.00) |  |
| Hepatic encephalopathy, n (%) |  |  | 1.000 |
| Yes | 0(0.00) | 0(0.00) |  |
| No | 5(100.00) | 5(100.00) |  |
| PVT, n (%) |  |  | 1.000 |
| Yes | 1(20.00) | 1(20.00) |  |
| No | 4(80.00) | 4(80.00) |  |
